# Somatic polyploidy is associated with the upregulation of c-MYC interacting genes and EMT-like signature

**DOI:** 10.18632/oncotarget.12118

**Published:** 2016-09-19

**Authors:** Alejandro Vazquez-Martin, Olga V. Anatskaya, Alessandro Giuliani, Jekaterina Erenpreisa, Sui Huang, Kristine Salmina, Inna Inashkina, Anda Huna, Nikolai N. Nikolsky, Alexander E. Vinogradov

**Affiliations:** ^1^ Latvian Biomedical Research and Study Centre, Riga, Latvia; ^2^ Institute of Cytology, St-Petersburg, Russian Federation, Russia; ^3^ Istituto Superiore di Sanità, Rome, Italy; ^4^ Systems Biology Institute, Seattle, USA

**Keywords:** c-MYC interacting genes, polyploidy, Warburg, stress, EMT

## Abstract

The dependence of cancer on overexpressed c-MYC and its predisposition for polyploidy represents a double puzzle. We address this conundrum by cross-species transcription analysis of c-MYC interacting genes in polyploid vs. diploid tissues and cells, including human vs. mouse heart, mouse vs. human liver and purified 4n vs. 2n mouse decidua cells. Gene-by-gene transcriptome comparison and principal component analysis indicated that c-MYC interactants are significantly overrepresented among ploidy-associated genes. Protein interaction networks and gene module analysis revealed that the most upregulated genes relate to growth, stress response, proliferation, stemness and unicellularity, as well as to the pathways of cancer supported by MAPK and RAS coordinated pathways. A surprising feature was the up-regulation of epithelial-mesenchymal transition (EMT) modules embodied by the N-cadherin pathway and EMT regulators from SNAIL and TWIST families. Metabolic pathway analysis also revealed the EMT-linked features, such as global proteome remodeling, oxidative stress, DNA repair and Warburg-like energy metabolism. Genes associated with apoptosis, immunity, energy demand and tumour suppression were mostly down-regulated. Noteworthy, despite the association between polyploidy and ample features of cancer, polyploidy does not trigger it. Possibly it occurs because normal polyploidy does not go that far in embryonalisation and linked genome destabilisation. In general, the analysis of polyploid transcriptome explained the evolutionary relation of c-MYC and polyploidy to cancer.

## INTRODUCTION

c-MYC is a potent, highly conserved transcription factor that interacts with at least several thousands of genes [1]. c-MYC can be considered as a pleiotropic sensor, integrating multiple cellular signals and mediating a transcriptional response that drives cell stress, growth/proliferation, and apoptosis. Activation of c-MYC transcription is an end-point for a broad range of signal-transduction pathways [2]. A link between c-MYC and initiation and maintenance of a wide range of neoplasms is well documented (for reviews, see [3–6]). However, c-MYC (and other members of its family) is rarely mutated in cancers but is activated by gene amplification or translocation, and the resulting abundance of c-MYC activity leads to cellular immortality associated with blockade of differentiation [2, 3]. c-MYC was discovered to act in synergy with another powerful oncogene, mutated (and constitutively active) RAS as a complementary pair in experimental murine tumors [7]. A tumor may critically depend on the activated c-MYC so that switching it off, as shown in transgenic mouse models, causes tumour regression. This phenomenon has been termed “oncogene addiction” [8]. c-MYC is also one of the Yamanaka factors used for induction of pluripotency in somatic cells (iPSC) [9].

In addition to having a large number of direct transcriptional targets, c-MYC is also a global amplifier of transcription [10] due to a wide range of secondary targets, resulting in a global increase in absolute cellular abundance of mRNAs [11]. It also causes chromatin remodeling to promote the more open conformation [8]. Importanly, overexpressed c-MYC causes endopolyploidy by decoupling DNA synthesis and mitosis [12].

Although somatic polyploidy (endopolyploidy) is normally encountered in a few normal mammalian tissues (liver, brain, vascular smooth muscle cells, heart, megakaryocytes, and placenta) [13–19], most solid tumours of any origin develop polyploidy and aneuploidy correlating with poor prognosis [20–27]. The tight association of malignancy with aneuploidy is a surprising fact in view of its essentially anti-proliferative effect [28–31]. Polyploidy came recently into the focus of cancer research because it can be induced in malignant cells (mostly with mutated TP53) by genotoxic agents. The reversed (de-polyploidised within one-three weeks) cells can serve as the origin of clonogenic recovery and resistance to anti-tumor drugs [14, 20, 25, 32, 33].

Because of this intricate relationship between c-MYC, polyploidy and neoplasia, we analysed the c-MYC interacting genes in normal polyploid cells to seek an answer for these two questions: (1) which properties of c-MYC that confer normal polyploidy may explain its function in promoting cancer and resistance to anticancer agents? (2) why normal polyploid cells are not tumorous and how they maintain normalcy? To this end, we analysed c-MYC-interacted genes associated with polyploidy from the available complete transcriptomes of liver, heart and placenta in mouse and human.

## RESULTS

### Polyploidy induces c-MYC interacting genes in heart, liver and placenta

To expose the evolutionary conserved ploidy-related functions among MYC-interacting genes, we compared the MYC interactomes (protein-protein interactions) in heart, liver and decidua cells. We took advantage of the patterns of pairwise reciprocal polyploid versus diploid organ comparison: human heart vs. mouse heart and mouse liver vs. human liver. The average nuclear ploidy in human liver is 2.05±0.008 n, whereas in mouse liver it is 5.47±0.1 n, and the average nuclear ploidy in mouse heart is 2.05±0.007 n, whereas in human heart it is 4.04±0.05n [34, 35]. Thus, human hepatocytes and mouse cardiomyocytes have predominantly diploid nuclei, whereas human cardiomyocytes and mouse hepatocytes have predominantly polyploid nuclei. Other authors also showed the higher ploidy level of mouse hepatocytes compared to human hepatocytes [36, 37, 38] and human cardiomyocytes compared to mouse cardiomyocytes [39, 40]. This reciprocal comparison across tissues and species removes species and tissue-specific signals and thus reveals evolutionary conserved ploidy-specific effects on gene expression (see also MATERIAL & METHODS).

The analysis was performed with three gene sets (as detailed in MATERIAL & METHODS): (1) the genes with common ploidy-associated change of expression in polyploid vs. diploid heart and liver and in tetraploid vs. diploid early mouse decidua cells (i.e. for three pair-wise comparisons); (2) the genes with similar direction of changes between polyploid vs. diploid heart and liver (i.e. for two pair-wise comparisons); (3) the genes with differential expression in tetraploid vs. diploid early mouse decidua cells (for one pair-wise comparison). Here we applied gene-by-gene and module-by-module comparisons. For better understanding of the functional relationships between genes, we constructed for these differentially expressed genes the corresponding protein interaction networks using the String database [1]. Finally, we verified the data on heart and liver obtained by NGS using the data obtained by microarrays [41].

We obtained three ploidy-associated gene lists containing 200 genes with increased and 76 genes with decreased expression for polyploidy versus diploidy in heart, liver and placenta (Supplementary Table S1). The corresponding numbers are 467 and 186 for ploidy-associated genes if only heart and liver are considered (Supplementary Table S2), and 1401 and 727 gens for the comparison in the 4n vs 2n early mouse decidua cells (Supplementary Table S3). The lists of biological modules significantly enriched for ploidy-associated genes with regard to the entire genome (13327 genes) and simultaneously to all known c-MYC interactants with known orthologs in human and mouse (3734) are presented in Supplementary Tables S4–S6. Although we focus only on the common heart, liver and placenta traits, we present here the gene and module lists for three gene sets. As can be seen from the Supplementary Tables S4–S6, the majority of polyploidy-upregulated genes in all three tissues belong to the modules related to growth, stress response, proliferation and stemness, including WNT, Pi3K, Hippo, Hedgehog, FGF, FOXM and TGF-beta (Supplementary Tables S4–S6) as well as protooncogenes that are supported by the MAPK system and belong to the Ras-coordinated gene module. This finding suggests a link between polyploidy and activation of fetal program and is in good agreement with the experimental data obtained with somatic cells reporting the ploidy regulation by Hippo signaling [42–44]. The most surprising common ploidy-associated feature of all three gene lists was the manifestation of epithelial-to-mesenchymal transition (EMT). In addition, we found features of fetal phenotype within the corresponding metabolic configuration, including the Warburg's effect.

Table 1 reports the genes with the strongest ploidy–associated regulation shared by the three data bases. It is evident that ploidy-associated genes include well-recognized major EMT regulators like SNAIL, BMP, N-cadherin as well as EMT metabolic marker stearoyl-CoA desaturase (SCD) [45]. The distribution of EMT-related genes among various functional groups associated with development (BMP2, SNAI2, BMP7), extracellular matrix and adhesion (CDH2, FN1; MMP14), metabolism (SCD) and sress response (EPAS1) as well as good gene concordance for different data bases suggests that EMT – is a polyploidy inherent feature. Some genes related to mesenchymal-epithelial transition (MET) are also upregulated, including HGF, WNT2B and FGFR1 (Table 1) and a few epithelial markers (Supplementary Table S1–S3) were revealed, as well.

**Table 1:** Ploidy associated Myc interacting core gene list for human and mouse heart, liver and 4n/2n decidual cells shared by 3 data bases ^a^^,^^b^

	Upregulated	Downregulated
	in myc-associated polyploidy	in myc-associated polyploidy
Cell Cycle	E2F8, CCNE1, CDKN2C, E2F5, CDK2, CCND3, LMNA,	CAV1, MYO5A, RB1, PAK2, RBL2, ARHGEF7 CDC42EP1, TBC1D4,
Growth and proliferation	BCAR1, EGFR, FGFR1, GNL3L, MYC, TK1, TLE1, MAP2K2, MCC, MTA1, HGF (MET), HRAS, NR2F2, RNH1, TAF6, TCF21, TGFA, IGFBP2, IGF1, WEE1, UBTF	RB1, PAK1; PAK2, RBL2
Development	WNT9B, GATA2, WNT2B (MET), AXIN2, DVL1, KIT, SNAI1 **(EMT), BMP2 (EMT),** FGFR1 (MET), **SNAI2 (EMT), BMP7 (EMT),**	
Matrix, cytoskeleton, and focal adhesion	CTTN, SDC4, TUBG1, DSTN, **FN1 (EMT),** NCAM1, **CDH2 (EMT), MMP14 (EMT),**	MEF2A, DSP, CYTH1, PLEK, MCMBP
Glutamine metabolism	GLS2, GCDH,	
Protein degradation	PSMA7, PSMB5, PEX11a	
Ribosome	RRS1, NOLC1	
Protein synthesis	GCAT,	
Sugar metabolism	PFKM, CA3, GNA11	CA2
Lipid metabolism	LDLR, PCBD1, **SCD (EMT)**	ALOX1
Tumor supressors	LATS1, TP53	RB1; PAK2; RBL2
DNA repair	TP53BP2, BRCA1, H2AFX	
Oxidative stress	**EPAS1 (HIF2A) (EMT),** PEX11, PEX16; HP	HP
Chromatin	HDAC11	
Signaling		NRIP1, TPST2, EXT1
Apoptosis		APAF1, BCL2L11, SYK, RB1, PAK, PAK2, RBL2 (p130)
Immunity		LCK, IL7, CD5, TNFSF13B, CD8A, CD22, NFATC2, IFIT2, MAML3, ZFAND5, HP

a Genes were obtained with databases [27, 32, 33]

b Gene functional categories have been chosen according to the GO classifications of the enrichment tools in String Data Base [1]. Genes may be present in more than one category.

(**EMT**) marks genes that were previously characterised as being induced or repressed, respectively, in epithelial-to-mesenchymal transition in the literature [34].

(**MET**) genes that were previously characterised as being induced or repressed, respectively at mesenchymal-to-epithelial transition in the literature [35]

### c-MYC and nucleostemin (GNL3L) demonstrate gene dosage exceeding induction in rodent tetraploid vs diploid hepatocytes

**Figure 1:**
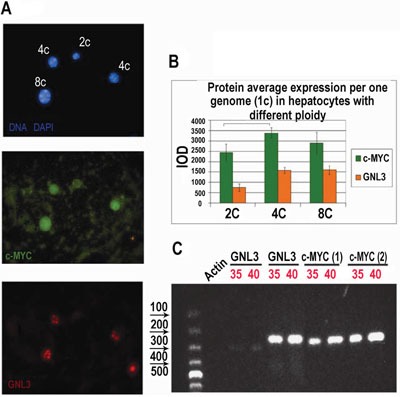
Immunofluorescent and RT-PCR study of polyploid versus diploid hepatocytes in mice **A.** Mouse hepatocytes were fixed and stained for c-Myc and nucleostemin (GNL3L) in combination with DAPI; **B.** Immunofluorescence image cytometry analysis showed that c-Myc and GNL3L protein content per genome is significantly higher in tetraploid cell nuclei as compared with diploid and **C.** RT-PCR performed with two different primer pairs, confirms expression of c-Myc and GLN3: 35, 40: PCR cycles number.

Cross-species and cross-tissue analysis of the c-MYC interacting genes revealed a higher c-MYC expression per genome in polyploid compared to diploid tissues and cells (Table 1, Supplementary Table S1). In particular, we found manifestation of stemness and induction of well-established stem-cell marker and direct target of c-MYC nucleostemin (GNL3L), which is normally not expressed in adult tissues [46, 47]. To confirm this finding, we carried out an immunocytochemical study of c-MYC and GNL3 protein content in polyploid vs. diploid hepatocytes of adult mice. We found that for both c-MYC and GNL3L protein content per genome is significantly higher in tetraploid cell nuclei than in diploid ones (in other words, it is higher than a gene dose). Interestingly, in octaploid nuclei the increased protein content per genome was not further elevated (Figure 1A, 1B). Similar results were obtained with adult rat livers (not shown). The characteristic elongated nuclei of Kupffer cells (liver macrophages) seen among hepatocytes served us as internal negative control for both proteins. Expression of c-MYC and GLN3L was confirmed by RT-PCR (Figure 1C). Our results suggest that cross-species data can be used to infer the intra-species relationships and that transition from diploidy to tetraploidy confers the cells the new properies linked to stemness and proliferative potential, which are not simply the result of increased gene dosage indicating to the change in transcription profile.

### Principal component analysis (PCA) identifies ploidy-associated conserved features: the significance of c-MYC

The main effects of c-MYC transcriptional regulatory activity are promotion of cell proliferation, embryonal programs, carbohydrate metabolism and protein synthesis [48, 49]. An important direct effect of c-MYC overexpression is the disconnection of DNA synthesis from mitosis, which results in polyploidy [12].

We next provide evidence for a potential function of c-MYC in polyploidy using a purely data-driven *a posteriori* approach. This strategy is based on the finding of enrichment of c-MYC interactants among the genes with significant scores (> 2SD) in the principal component axis related to ploidy. In addition to confirming of c-MYC involvement in ploidy, the approach gives global metabolic characterization of ploidy. Table 2 presents the loading pattern and the percentage of explained variability of the principal components of the heart-liver data set. PCA shows a clear hierarchical order of relevance in terms of explained variation and consequently of the associated biological factor:

**Table 2:** Loading pattern for heart and liver

	PC1	PC2	PC3	PC4
HS*_heart	0.691	0.529	−0.390	−0.301
HS_liver	0.690	−0.539	−0.366	0.313
MM**_heart	0.650	0.583	0.394	0.286
MM_liver	0.670	−0.556	0.398	−0.290

* Human;

** Mouse

PC1 (45.6% of total variance) corresponds to Shared Variability: more-to-less expressed genes independent of species, tissue and ploidy (all the variables enter PC1 with loadings of the same sign). This component probably reflects the ‘house-keeping’ gene fraction, which can be considered a ‘size’ component [50].

PC2 (30.5% of total variance): corresponds to Tissue-Effect: heart and liver samples enter the component with opposite loadings; PC2 is thus a ‘shape’ component [50] linked to the differential profiles of the two tissues. High values of component scores point to genes with higher expression in the heart than in the liver, while the opposite holds for low component scores (note that the loadings correspond to the correlation coefficients between variables and components).

PC3 (15% of total variance): corresponds to Species-Effect: mouse and human samples enter PC2 with opposite loadings irrespective of the tissue type. High values of component scores correspond to genes whose level of expression is higher in mice than in humans, while the opposite holds for low values of components.

PC4 (9% of total variance): corresponds to Ploidy-Effect: polyploid samples (HS heart and MM liver variables) have negative loadings on PC4 while diploid samples (MM heart and HS liver) show positive loadings. This implies that high component scores correspond to genes whose expression is suppressed by polyploidy condition, while low component scores correspond to genes whose expression level is increased by polyploidy as such.

It is worth noting that the fourth component could in principle only represent the ‘noise’ component given we have an initially four dimension space. So we checked for the non-gaussian (and consequently non-noisy) character of PC4. The signal character of PC4 was confirmed by its huge Kurtosis value (502.04 to be compared to the value of 3 typical of normal distribution) pointing to the fact the by far the major portion of PC4 variance was accounted for few outlier genes. The soundness of 2SD threshold was confirmed by the fact the 99% percentile of PC4 distribution (that has by construction 0-mean and unit standard deviation) is at 1.38SD (another proof of concept of its non-gaussian character).

The result shows that polyploidy does not exert dramatic effect on transcriptome and accounts for only about 10% of variability in the human vs. mouse heart as well as in the mouse vs. human liver. PCA results are in agreement with generally accepted notion that the effects of polyploidy are weak and unique because of gene dosage compensation for the majority of genes [51]. At the same time, PCA revealed a minor albeit significant pure ‘polyploidy-related’ component independent of both species and tissue–linked effects.

Notably, we extracted four components starting by an initially four-dimensional system; this implies that we applied PCA as a pure geometrical transformation corresponding to the rotation of the initial data set into a basis set spanned by mutually orthogonal axes (components) with no loss of information. The hierarchical character of component extraction (the components are numbered in decreasing order of variance explained) reflects the relative importance of shared (house-keeping genes), tissue, species and ploidy effects. The expression value of each gene can be comprised as a summation over the four components. Given that each component has by construction zero mean and unit standard deviation over the whole set of genes we can consider the genes having a PC4 score exceeding 2 SD (in a module) as the genes exceeding the 95% confidence interval with respect to PC4 and thus, ‘significantly’ contributing to the PC4 ploidy component [52]. To investigate whether PCA revealed ploidy associated genes (we considered genes having a score > 2 SD in a module) are enriched for c-MYC interactome, we extracted c-MYC interactants from the String database [1]. After that, we matched them to all 13327 human-mouse orthologous genes. Overall, we obtained 3327 genes, which make up a proportion of 0.24 of all orthologous genes. Using binomial test for comparison of this proportion to the proportions of c-MYC interactants among significantly ploidy-induced genes, 0.408 (55 of 134), and ploidy-inhibited genes, 0.5010 (50 of 98) (Supplementary Tables S7 and S8) show a p-value < 0.000005 for the difference between 0.24 and 0.408 and p< 0.0000001, for the difference between 0.24 and 0.501, respectively.

**Figure 2:**
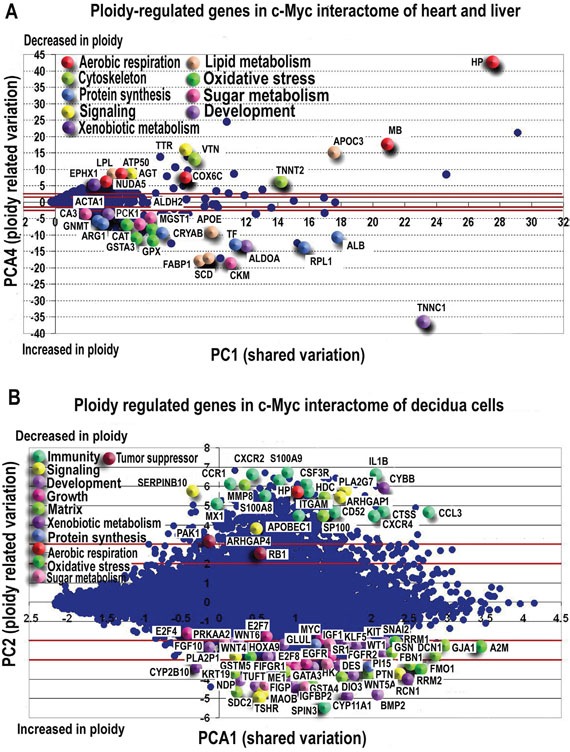
PCA revealed ploidy-associated genes in c-Myc interactome of heart-liver A. and placenta B. Genes demonstrating the most pronounced variation with ploidy are indicated by enlarged symbols Symbol colours represent gene functions as listed in legend. In both, heart-liver (A) and in placenta (B) polyploidy is associated with the induction of developmental markers and genes related to protein synthesis, oxidative stress response and sugar metabolism. Genes related to aerobic respiration are mainly repressed. Purple lines mark 2SD and 3SD.

Figures 2A and 2B present the MYC-interacting gene distribution in PC1 of PCA space, with the 2 and 3 Standard Deviations lines on PC4 shown. The strong coherence between two completely different statistical paradigms of selection (PCA does not explicitly encompass ploidy in the algorithm being only based on the between profiles mutual correlations) offers a proof-of-concept of the robustness of the obtained results. This coherence allows us further sketch the functional description of c-MYC interactants. We base our analysis on values of genes scoring in PC4 higher than three SDs together with the major known ploidy-regulator genes with a lower than 2 SD PC4 scores.

In this way, we found that C-MYC interacting genes with substantial ploidy variation participate in cytoskeleton maintenance, growth, ATP reservation, energy metabolism and oxidative stress protection (Figure 2A, Supplementary Table S7). GO categories and KEGG pathways enriched in ploidy-associated genes with more than 2 SD expression difference between polyploid/diploid organs and cells (Supplementary Table S8) relate to oxidative and xenobiotic stress response, protein synthesis and processes related to single cell organisms.

To identify ‘ploidy-related’ effect on gene expression in purified diploid and tetraploid mouse decidua cells, we applied the same geometrical approach.

In this case, we have a bidimensional initial data set corresponding to two expression vectors of diploid and tetraploid cells (See Supplementary Table S9). The complete PCA solution gives rise to a two components space spanned by a pre-dominant shared variation axis (size component, correspondent to the cell developmental differentiation attractor state) in which diploid and polyploid tissues are loaded with the same sign and a minor ‘ploidy’ component encompassing the divergent expressions between the two conditions (the two vectors enter with opposite loading). Table 3 shows the correspondent loading pattern:

**Table 3:** Loading pattern for decidua cells

	PC1	PC2
Diploid	0.981	0.193
Tetraploid	0.981	−0.193

As expected, PC1 explains the major part of variability (96.3%), it shows the existence of a very strong and invariant ‘tissue attractor’ correspondent to the specific placental expression profile (see for example [53]), while ploidy related PC2 accounts for a minor portion of expression variation (3.7%).

Nevertheless, such minor variation allows for the identification of some relevant genes: in Supplementary Table S9 the ‘relevant’ gene expressions (higher than 2 or lower than −2 SD units) introduce in function-related evidence. Consistently with the loading signs, (see Supplementary Table S9) positive values of the component correspond to genes whose expression is higher in diploid state, while negative values correspond to genes whose expression is higher for polyploid tissues. Figure 2B presents this situation. X- axis corresponds to the PC1 shared variation and Y axis to ploidy factor, two lines are set at the 2 SD thresholds.

To examine whether c-MYC interactants are significantly over-represented among the genes of decidua cells revealed by PCA as tetraploidy related, we matched them to all 22020 genes of this set. The obtained 3845 genes comprised 0.19 of all genes. Then, like for heart and liver, we compared this proportion to the proportions of c-MYC interactants among significantly ploidy-induced genes 0.348 (152 of 436) and ploidy-inhibited genes 0.292 (242 of 828) using binomial test. The results show a near zero p-value (p<10^−24^) for the difference between 0.19 and 0.348 and a p-value = 7.79821E-13 for the difference between 0.19 and 0.29.

**Figure 3:**
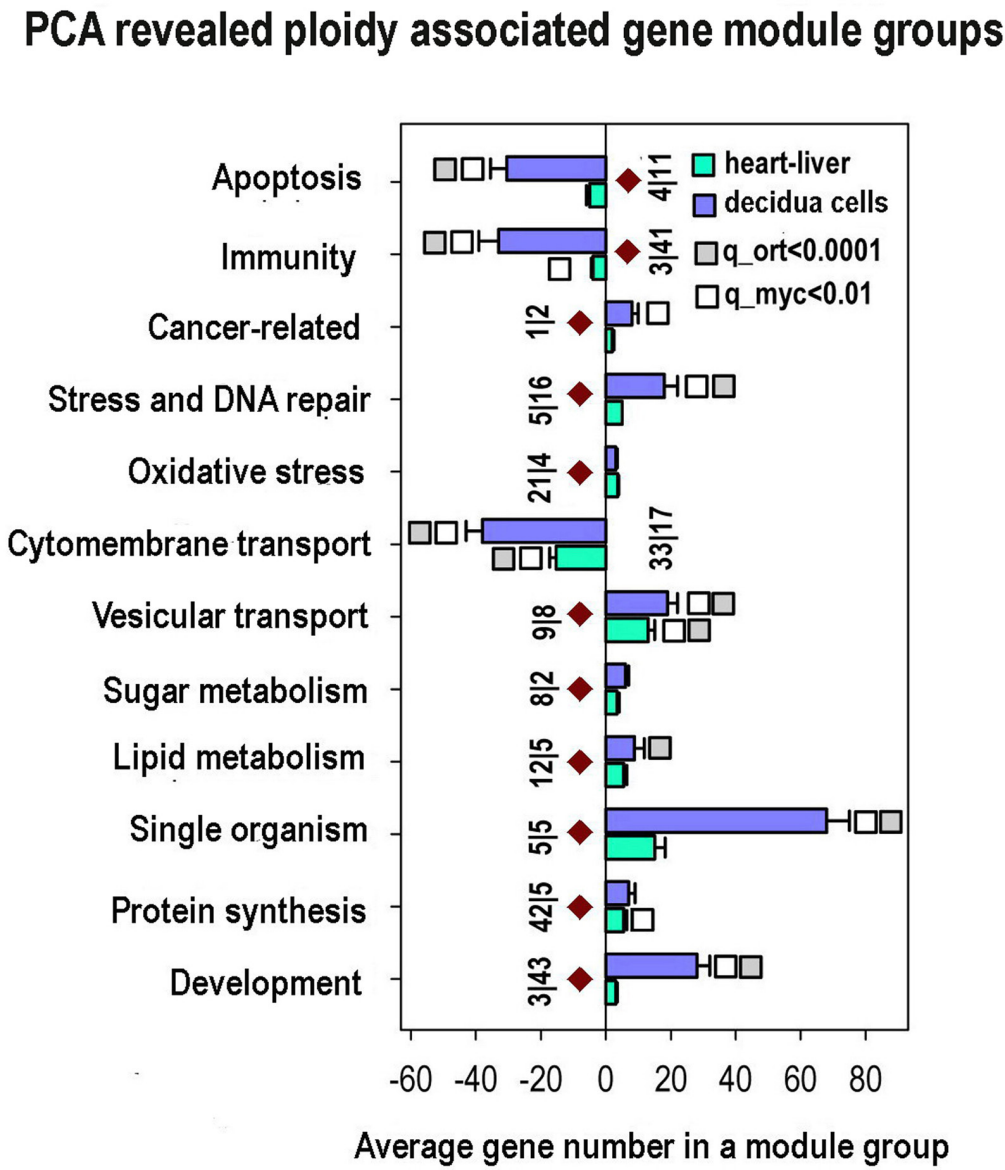
Common ploidy associated changes in gene functional module groups for heart-liver and 4n/2n decidua cells revealed by PCA X axis - average gene number in a module group. Y axis - module functional group names. Figures at the bottom of the bars indicate module number in a functional group. Small vertical bar divides figure for heart-liver and for 4n/2n decidua cells. White and grey squares reflect geometrical mean for q values of module functional group enrichment significance with regard to all Myc targets (white squares) and with regard to all orthologs (grey squares). Bars with no squares have q-value not less than 0.15 with regard to all Myc targets and not less than 0.05 with regard to all orthologs. Module groups confirmed by gene-by-gene analysis are marked with brown diamonds.

To present briefly the ploidy-related effects revealed by PCA in early mouse decidua, we describe the up- and down-regulated c-MYC interactants with the most prominent expression difference between diploid and tetraploid cells. We also specified the most important biological regulators demonstrating significant variation with ploidy. Gene function description is in Supplementary Table S9. Finally, Figure 3 shows the MYC-interacting gene distribution in PC1-PC4 space for polyploidy effects of three tissues with lines indicating 2 and 3 Standard Deviations on PC4. We also provide below a brief functional description of the c-MYC interactants displaying more than 3 SD and several principal biological regulators with lower than 3 SD but significant ploidy-regulation.

The analysis of biological modules enriched in ploidy-associated genes in heart, liver and in decidua cells using PCA (Figure 3) indicates that polyploidy activates a response to oxidative stress, DNA repair, and modules related to cancer, thus suggesting that genome duplication enhances oncogenic proclivity. As well, we found clear manifestations of the fetal program significant for cancer [54] including the induction of modules related to single cell organisms, protein synthesis and modules regulating sugar and lipid metabolism. In accordance with oncogenic and fetal traits, modules of apoptosis and immunity are inhibited (Figure 3). Modules of transport show the downregulation of cytomembrane transport and upregulation of vesicular transport. This modification is in agreement with ploidy related decrease of cell surface to volume ratio and with its compensation by active vesicular transport [21–23]. Importantly, practically all changes revealed by the PCA module groups are in a good agreement with gene cross-species comparison. These modules are marked in Figure 3 with brown diamonds.

In summary, PCA of gene expression profiles in heart-liver and 4n/2n decidua cells revealed the following biological features of gene expression programs associated with polyploidy: response to oxidative and xenobiotic stress, embryonality, apoptosis impairment, the shift to anaerobic and ATP saving type of energy production, and induction of modules related to single cell organisms.

### Ploidy associated protein interaction networks reveal synergetic activation of regulome, embryonic features, and stress response

**Figure 4:**
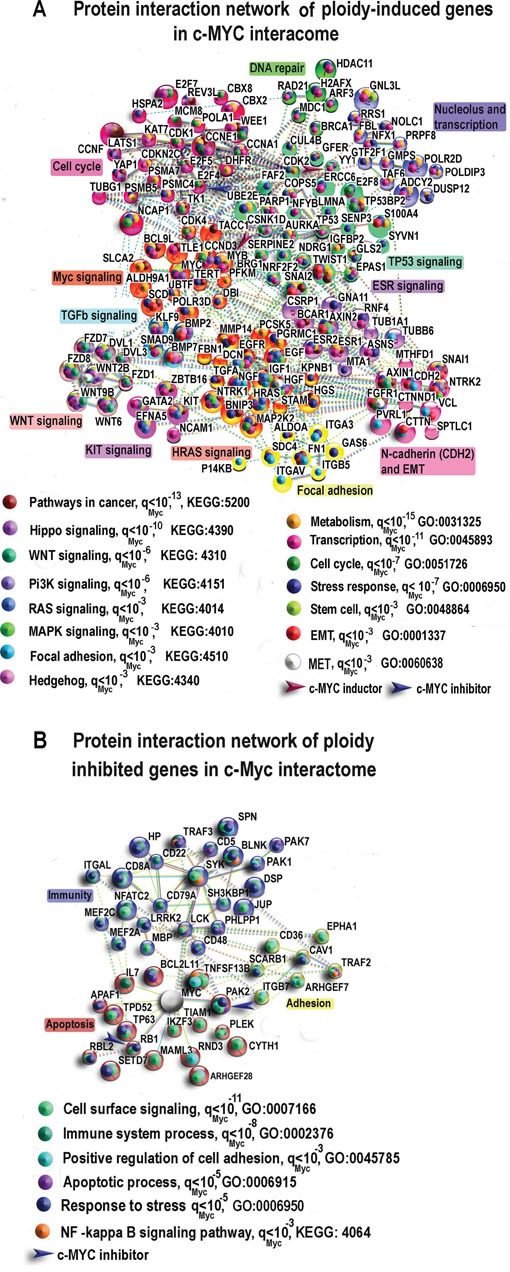
The most connected components of protein interaction networks of significantly ploidy- induced A. and ploidy-inhibited B. genes in the c-Myc interactome of heart, liver and placenta revealed by gene-by-gene cross-species comparisons of human and mouse heart and liver and 4n/2n mouse decidua cells Large symbols show genes with more than two-fold expression differences between polyploid vs diploid organs and cells. Brown and blue arrows show direct c-MYC inductors and inhibitors that were determined with the use of String Server (molecular interaction type option). Clustering was performed by MCL algorithm with the use of the same server. qMyc presents q value for GO biological processes and KEGG pathways enrichment of tested gene sample compared to all c-Myc interactants.

Modules and protein interaction networks can offer a link between genes and biological functions, thus consitute a key step in connecting genotype and phenotype [55]. Therefore, we next constructed protein interaction networks encoded by the genes positively and negatively related with polyploidy with a high stringency for interaction (>0.9). Such 'ploidy induced' network containing clusters of c-MYC, p53, cell cycle, WNT, HRAS, IGF signaling, nucleoli and extracellular matrix is presented in Figure 4A. The protein network of 'ploidy-repressed' genes presented in Figure 4B contains clusters of inflammation, lipid metabolism, tumor suppression, and apoptosis. As can be readily seen, the induced network contains more transcription factors, multifunctional regulators and growth factors (E2f 4, 5, 7, 8, SNAI1, 2, TWIST1, HRAS, c-KIT, c-MYC, GATA2, TP53; WNT6, 2B, 9B, BMP2, 7, IGF1, EGF, EGFR, HGF, FGFR1) than the ploidy-inhibited network (RB1, PAK1, PAK2, PAK7, MEF2a, c, APAF1). Accordingly, GO biological processes and KEGG pathways in the polyploidy-induced network are related to cancer, metabolism, cell cycle, stem cell pathways (Pi3K, Hippo, Hedgehog, WNT), EMT and MET pathways and stress response (Figure 4A), while again, the pathways of apoptosis, cell death, inflammation and cytoskeleton with Rho signaling elements are inhibited (Figure 4B). To find out whether the association between polyploidy and c-MYC is reciprocal, we also analysed the types of molecular interactions for the networks depicted at Figure 4A and 4B using server String. Our data indicate that polyploidy influences several genes targeting c-MYC via direct binding (Figure 4A and 4B). The ploidy-activated genes include c-MYC inducer YY1 [56], oncogene MYB [57], and E2F4 which retards c-MYC increased proliferation via negative feedback loop in the mitotic restriction (R) point [58]. The genes inhibited by ploidy are also presented by c-MYC suppressors RB1 [59] and PAK2 [60, 61]. At the same time, it is established that c-MYC overexpression uncouples DNA replication from mitosis completion causing polyploidy as such [12, 62, 63] and our study of cell cycle regulating genes confirmed it (see below). The causal relationship between the overexpressed c-MYC and normal polyploidy was clearly confirmed in mouse hepatocytes where overexpressed or underexpressed c-MYC correspondingly accelerated or retarded developmental polyploidization [64, 65]. So, in general the data suggests that the programmed overexpression of c-MYC causing developmental polyploidy is also under some feedback control by it.

To investigate the data obtained from the bird's eye in more details, we performed the manual data curation and analysis of gene modules related to specific functions briefly described below.

### Cell cycle regulation reveals polyploidy-associated proliferation potential

**Figure 5:**
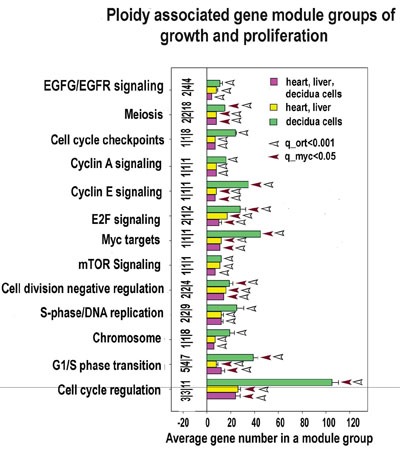
Proliferation and growth related modules significantly enriched in ploidy-regulated genes X axis - average gene number in a module group. Y axis - module functional groups. Figures at the bottom of the bars indicate module number in a functional group. Small horizonatl bar divides the figures for heart-liver, for decidua cells and for heart, liver and decidua cells. Red and white arrows reflect geometrical mean for q values of module functional group enrichment significance with regard to all Myc targets (red arrows) and with regard to all orthologs (white arrows). Bars with no squares have q -value not less than 0.15 with regard to all Myc targets and not less than 0.05 regarding all orthologs.

**Figure 6:**
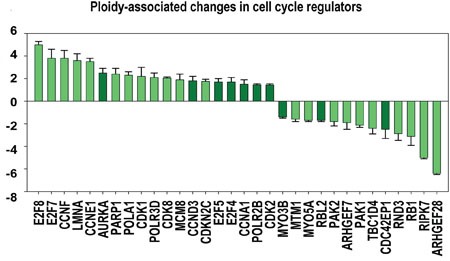
Ploidy-associated changes in the activity of regulators related to cell cycle X- gene names; Y-average expression for heart-liver and placenta±SE. Bars of light colors correspond to p<0.0001; Bars of dark colors correspond to p<0.01. This chart shows the increased activity of cell cycle regulators related to G1-S transition (CCNA1, E1, D3, F; CDK2, 8; E2F4, 5,) S-phase (POLA1, PARP1, REV3L, MCM8, TK), polyploidization (E2F7, 8) G2-M genes (AURKA1) and decreased activity of genes involved in cytokinesis (ARHGEF28, ARHGEF7, CDC42EP1, MTM1, MYO5A, MYO3B, RIPK7, CAV1) and tumor supressors (RB1, RBL2, PAK1, PAK2).

Polyploid hepatocytes and cardiomyocytes were reported arising via aborted (polyploidising) mitoses (reaching ploidies 4-8C, rarer 16-32C) [17, 37, 66]. Our data are fully in agreement with these observations (Figures 4A, 5, 6 and Supplementary Table S4–S6). They reveal features of G1-S induction (Cyclines A1, E1, D3, CDK2, 8, MCM8, TK1, POLA1, PARP1, REV3), metaphase entry (AURKA1 activation), polyploidization (E2F7, 8), and cytokinesis omission (inhibited Rho signalling and cytoskeleton elements ARHGEF28 and ARHGEF7, CDC42EP1, MTM1, MYO5A, MYO3B) coupled to senescence suppression (inhibited PAK1, 2, 7). Thus, we confirm that normal polyploid cells originated by aborted cytokinesis represent in fact a reservoir for cell division and growth [17, 66].

**Figure 7:**
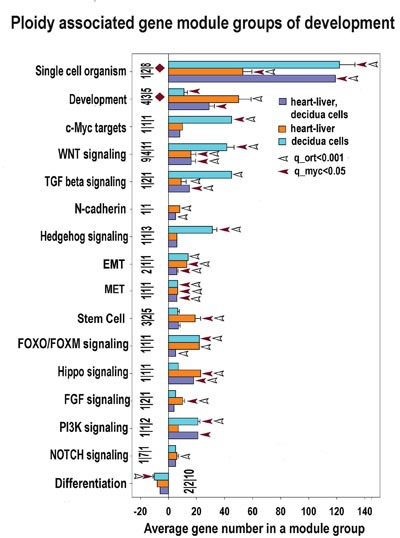
Development and stemness modules significantly enriched for ploidy-regulated genes from all three c-Myc interacting gene lists (for heart-liver, placenta and heart-liver-placenta) Designations for X, Y, figures at the bottom of the bars, red and white arrows pointing to the tips of the bars, bars with no arrows and red diamonds are the same as for Figure 5. This chart indicates that polyploidy is linked with metabolism activation and modification. This chart demonstrates common nature of ploidy-related stemness and the induction of epithelial-to -mesenchymal transition. The stemness is seen from the upregulation of modules related to stem cell and signaling by PI3K, NOTCH, HIPPO, FGF, FOXO/FOXM WNT, TGF-beta, c-MYC, Hedgehog. Activated epithelial-to -mesenchymal transition is evident from the activated EMT module.

### c-MYC activation of the ancient Wnt and TGF-beta pathways is associated with the EMT-featured properties of polyploidy

**Figure 8:**
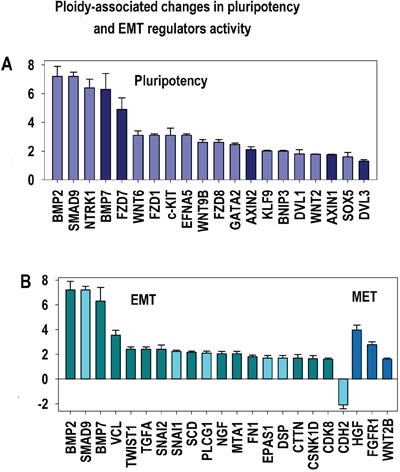
Ploidy-associated regulators of epithelial-to-mesenchymal transition (EMT) and pluripotency X- gene names; Y-average expession for heart-liver and placenta±SE. Bars of light colors correspond to p<0.0001; Bars of dark colors correspond to p<0.01. This chart shows the increased activity of principal regulators related to selfrenewal (A) and EMT (B) increasing ploidy proclivity for transformation.

Our data in all three gene lists show a clear transcriptional activation of GO modules related to Wnt pathways playing a prominent role in controlling cell fate decisions during embryonic development (Supplementary Tables S4–S6, Figures 4A, 7, 8). As well, the genes involved in Wnt pathways regulation were clearly ploidy-upregulated and form a tight subnetwork (Figure 4A). In concordance, we identified the induction of WNT cross-regulated pathways related to transformation (IGF, mTOR, HGF, RAS, E2F (Figures 7, 8) and stemmness (the pathways related to pluripotency, stem cell biology, and Hedgehog, NOTCH, PI3K, FGF, Hippo and TGF- pathways) (Figure 7, Supplementary Tables S4–S6).

Our observation of an increased activity of WNT-TGF beta signaling is in a good agreement with the general activation of epithelial-mesenchymal transition (TWIST1, SNAI1, SNAI2, VCL, TGFA, FN1 (Fibronectin) and CDH2 (N-Cadherin) (Figures 4A, 8, Table 1, Supplementary Tables S1–S3).

All these facts suggest that c-MYC-related activation of the WNT/TGF beta pathways is a key component of the ploidy-associated network with the tumour-like properties including stemness and EMT. At the same time, coordinated induction of EMT– related genes is coexisting in all three gene lists with a few genes participating in MET that reverse EMT and with some epithethelial markers (Supplementary Tables S1–S3).

### c-MYC-associated common metabolic profiles of polyploid cells from heart, liver and placenta

Ploidy-related changes in macromolecule metabolism show enhanced transcription activity, ribogenesis, highly dynamic protein turnover, global proteome remodeling and activated lipid metabolism.

**Figure 9:**
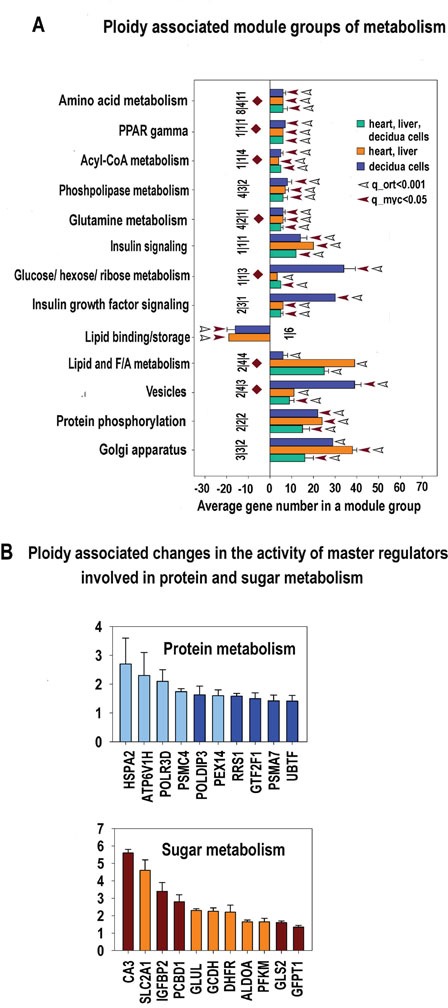
**A.** Metabolism related modules significantly enriched in ploidy-regulated genes from all three c-Myc interacting gene lists (for heart-liver, placenta and heart-liver-placenta). Designations for X, Y, figures at the bottom of the bars, red and white arrows pointing to the tips of the bars, bars with no arrows and red diamonds are the same as for Figure 5. This chart indicates that polyploidy is linked to metabolism activation and modification. Specifically, it outlines the boosting of various branches of protein metabolism and transport, induction of sugar metabolism and insulin signaling and the switch of the lipid metabolism from biosynthesis to decomposition. This switch is seen from impaired modules of lipid binding and storage and induced modules related to phospholipase, Acyl-CoA metabolism and PPAR gamma modules. **B.** Ploidy-associated metabolic regulators common for heart-liver and placenta. X- gene names; Y-average expession for heart-liver and placenta±SE. Bars of light colors correspond to p<0.0001; Bars of dark colors correspond to p<0.01. This chart shows the increased activity of sugar and protein metabolism inherent for increased activity of growth processes.

The metabolic genes and gene modules demonstrating similar ploidy-associated changes in heart, liver and early mouse decidua are presented in Supplementary Tables S1–S6 and in Figure 9. The main functions of the upregulated gene modules are positive regulation of protein metabolic process related to protein transport and phosphorylation. These findings suggest that the proteomic landscape of polyploid cells is very active and differs from that of diploid cells. The upregulation of the phosphate- and phosphorus-related metabolic modules, which are also involved in protein modification and/or cellular signaling regulation, is in agreement with the notion of a global and dynamic proteome remodeling of polyploid cells (Supplementary Tables S3–S5).

This highly dynamic proteomic profile implies both protein degradation and synthesis. Therefore, our data highlights a specific upregulation of genes involved in the lysosomal and proteasomal protein degradation (Supplementary Tables S1–S6) and the upregulation of mTOR pathway (Figure 5, Supplementary Tables S4–S6) that enhances translation efficiency and ribosomal biogenesis [67].

In addition, a switch of lipid metabolism from biosynthesis to decomposition was revealed from impaired modules of lipid binding and storage and induced modules related to phospholipase, Acyl-CoA metabolism, and PPAR gamma modules. This result is in agreement with the recent data by Edmunds and colleagues [68] evidencing increase of fatty acid utilization by c-MYC activation.

All this shows the highly dynamic features of polyploid cells showing enhanced transcription, ribogenesis, protein turnover, and lipid metabolism.

Stress response: DNA synthesis and repair machinery, cellular detoxification, protection against oxidative stress and protein glycosylation

**Figure 10:**
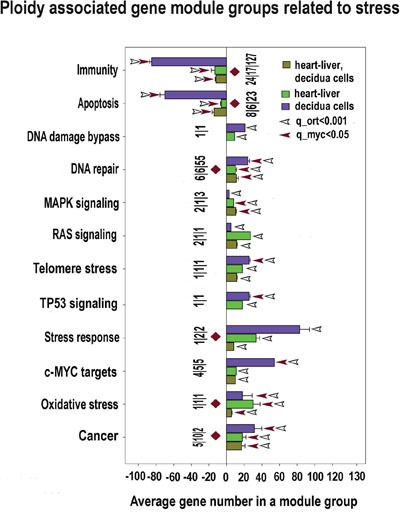
Stress response and transformation related modules significantly enriched in ploidy-regulated genes from all three c-Myc interacting gene lists in all three comparisons (for heart-liver, placenta and heart-liver-placenta) Designations for X, Y axes, figures at the bottom of the bars, red and white arrows pointing to the tips of the bars, bars with no arrows and red diamonds, and the method of data obtaining are the same as at Fig 5. This chart demonstrates coordinated induction of module groups related to stress, DNA repair and transformation (cancer-related module group and module groups of c-Myc, RAS and MAPK) and the down-regulation of module groups related to apoptosis and immunity.

**Figure 11:**
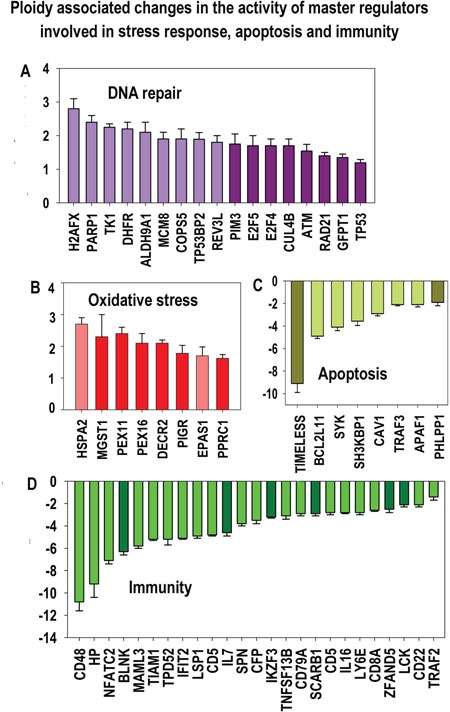
Ploidy-associated changes of master regulators activity invoved in DNA repair, oxidative stress, apoptosis and immunity common for heart-liver and placenta X- gene names; Y-average expession for heart-liver and placenta±SE. Bars of light colors correspond to p<0.0001; bars of dark colors correspond to p<0.01. This chart demonstrates a combination of induced stress response and suppressed apoptosis and immunity that may increase the risk of cell transformation.

Our data indicate that polyploidy is associated with adaptation to stress. This is evident from the activated pathways crosstalk and general transcriptome elevation (Supplementary Tables S1–S6; Figures 10, 11). The increase of the genome dynamic is seen from the prevalence of induced genes and gene modules over inhibited ones (Supplementary Tables S1–S6; Figures 10, 11) and from a larger (having larger number of connected genes) protein-protein interaction network for the induced genes than for the inhibited ones (Figure 4). Notably, in accordance with the transcriptome activation, all clusters presented in the network on Figure 4 are by 40-90% composed of the genes implicated in stress response (besides their basic functions). Among various stress-related branches, the highest significance of induction (confirmed by PCA) is exposed by the genes involved in DNA repair, oxidative stress and cancer (Figures 3, 10, 11).

Other upregulated genes are involved in detoxification and protection of cells from the oxidative stress induced during catabolism of amino acids and carbohydrates, such as ALDH9A1, CA3 and PIM3. Notably, an increased aldehyde dehydrogenase activity, along with an increased expression of c-MYC and activation of the WNT/β-catenin, is a feature of cancer stem cells [69].

We also observed an enhancement in the expression of glutamine-fructose-6-phosphate transaminase 1 (GFPT1), the gene involved in the channeling of glucose flux into hexosamine pathways. Notably, the global protein glycosylation level has been reported being increased upon c-MYC activation and elevated in cancer cells [70].

Interestingly, the response to apoptosis and immune-related modules were coordinately downregulated (Figures 3, 10, 11), in line with the data on protein network presented in Figures 4B.

Glycolysis and glutaminolysis are the main sources of carbon and energy

Polyploid cells supporting highly biosynthetic metabolism need a source of energy and carbon supply to provide ATP and building blocks for DNA and protein synthesis. Notably, the c-MYC oncogene was found as playing a major role as a central organizer of the metabolic changes, which occur in transformed cells [12, 49].

Our transcriptional metabolic analysis discloses that the main nutrients used by polyploid cells seem to be carbohydrates and aminoacids. Thus, we observed an enhancement of the levels of expression of the enzymes and modules involved in glucose, fructose and mannose and glutamine metabolism (Figure 9, Supplementary Tables S4–S6, S9 and S10), also known as a characteristic feature of cancer cells. In heart and liver this is combined with down-regulation of oxidative phosphorylation (Warburg effect), while mitochondrial respiration is high in placenta.

As a summary of metabolome analysis, we conclude that the c-MYC-related metabolism of polyploid cells shows elevated protein turnover (synthesis and degradation) that employs carbohydrates and aminoacids as carbon and energy source. Thus, these polyploid cells not only express several EMT markers but also present the EMT-consistent metabolic features.

### RAS – a complementary partner of c-MYC for oncogenesis is enhanced and creates a hub in the polyploidy activated c-MYC interacting genes

Studies on collaboration of two powerful oncogenes, c-MYC and RAS, have provided one of the basic concepts of carcinogenesis occurring in two steps ‘immortalisation/initiation” (amplified c-MYC) and transformation/promotion (mutated RAS) [7]. However, the complementation of c-MYC and RAS, which is sufficient and effective for full carcinogenesis as found in early studies still remains poorly understood [71, 72]. Therefore, up-regulation of the oncogenic module of MYC/RAS in polyploidy associated network deserves particular attention (Figure 4A).

Both oncogenes are linked to stress response (first of all by JUN and FOS, AP-1 complex with c-MYC) via conservative MAPK pathway and they directly activate and stabilize each other [54]. More recent studies show that both RAS and c-MYC are most often induced from EGFR by EGF and TGFα (found here induced by polyploidy 6-, 4- and 2-fold, correspondingly) and converge on Cyclin D/Cdk2 (also induced) activating proliferation, where immortality may be supported by c-Myc [65].

In view of the carcinogenic potential of MYC/RAS complementary pair revealed here in normal polyploidy cells, we undertook a more detailed study of the c-MYC related tumour suppressor TP53 interacting genes.

### TP53 and malignancy traits

The tumor suppressor TP53 is a central coordinator of the adaptive cellular response to stress conditions that facilitates repair and survival of damaged cells or eliminates severely damaged cells [73–75], and most importantly, it is a main barrier of cells to cancer [54]. TP53 is enhanced in all three polyploid tissues, however only slightly (16%), while the down-stream CDKN1A/p21CIP1 (with its positive feedback to p53) is not activated, in spite of the up-regulation of the DNA damage response genes (Supplementary Table S1, Figure 4A). Suppression of senescence, apoptosis, and the relatively modest upregulation of p53 may suggest a lowered barrier of polyploidy cells to genome instability and malignancy. Moreover, c-MYC overexpression can compromise and override some of the TP53-dependent responses activated by cellular stress changing thereby the global cellular effects of TP53 activation [76]. In turn, tetraploidisation of cancer cells surpasses the effect of downregulation of p53 in their diploid conterparts as judged by survival in response to oxidative stress [77].

### Summary of results

**Figure 12:**
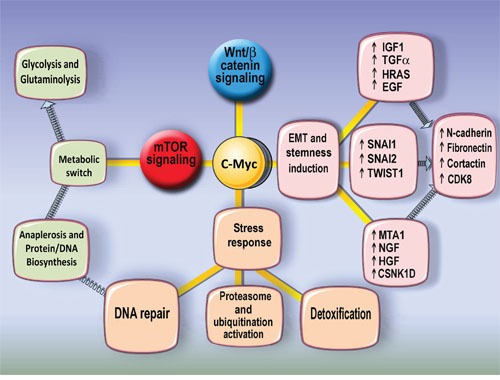
The scheme illustrates the main features of polyploidy-associated Myc interacting genes upregulation The upregulation of EMT and stemness master regulators as well as metabolic switch to glycolisis and glutaminolysis and enhanced protein synthesis and stress response pathways may suggest that polyploidy increases addiction to transformtaion.

We conclude that c-MYC- related polyploidy favours the expression of cellular programs of malignancy-related pathways (TGFb/WNT, BMP/WNT-embryonality, EMT, stress response, DNA synthesis and repair, Warburg-type energy supply, and activation of complementary proto-oncogenes). The c-MYC related metabolome of polyploid cells supports stress response and energy saving pathways coupled to EMT (Figure 12).

## DISCUSSION

Nothing in Biology Makes Sense Except in the Light of Evolution (T. Dobzhansky)

### c-MYC-related attraction of polyploidy to cancer represents an evolutionary toolkit for adaptation to stress

We have undertaken this research to address two questions: (1) which properties of c-MYC confers polyploidy that may explain its role in promoting cancer; (2) why normal polyploid cells are not tumorous. Upregulation of c-MYC in polyploid cells of mammalian tissues has already been reported [12, 65], however c-MYC-interaction gene and protein network in polyploid cells have not been systematically analysed at a genome scale. PCA approach applied in our study, firstly, confirmed the fidelity of main results obtained by cross-species reciprocal comparison summarised above and, secondly, highlighted its evolutionary aspect. The found features of c-MYC related polyploidy, such as enhanced DNA repair, replication, and development, illustrate the adaptive and driving role of polyploidy in evolution, confirmed by mutual pathways with the whole genome duplications [78–80]. These adaptive features can explain 'addiction' of polyploidy to cancer. Moreover, PCA clearly exposed a single-cell organism module addressing origin of somatic polyploidy to the transition period from unicellular to multicellular organisms, which has occurred about 600 mln years ago [81, 82]. Segregation of germ and soma, division of labour, and gastrulation (movement of cell masses with EMT as its component [46]) were the first acquisitions of early multicellularians

Therefore, it is worth noting that the c-MYC related TGFβ/Wnt pathways interacting with genes of the EMT program found here as playing a central role for normal somatic polyploidy are activated in embryogenesis from the gastrulation stage on. Thus, we revealed that normal differentiated cells developmentally polyploidised through abortive mitosis by c-MYC overexpression [12, 64, 65] become also embryonalised and stress-responsive by it. Obviously, by origin this program represents an evolutionary toolkit for adaptation to stress. These evolutionary traits of transient polyploidy linked to embryonalisation and exploiting c-MYC likely became usurped by cancer cells [83–87] conferring them resistance to treatments coupled with proliferative and metastatic potential. It appears that adaptive advantage of tetraploidy trades off the proliferative disadvantage of inevitable aneuploidy, explaining the “aneuploidy paradox” in cancer cells. Interestingly, studies by Duncan and colleagues showed that tetraploid mouse hepatocytes, when isolated and cultured display, contrary to wild type possessing normal karyotype in ~99% of cells, a high proportion of chromosome missegregations [37, 88]. Thus, in very stressful conditions, the genome of normally polyploid hepatocytes is prone to instability. In turn, genome instability as such promotes tumorigenicity [89]. Therefore, by all reasons, killing preneoplastic tetraploid cells is a useful strategy for cancer chemoprevention [90].

### However, why is normal polyploidy related to cancer but does not always cause cancer?

The proclivity of c-MYC-polyploidy associated genes towards cancer contradicts the absence of active proliferation, genome instability, and cancer in these normal tissues. Notably, the shift towards embryonality of normal polyploid cells, which we have revealed and explored, remains in the developmental realm of gastrulation embryo. In spite of overexpressed c-MYC, which is tempered by wt TP53 and some negative feedbacks, it does not reach the pluripotent embryonal stem cell state (manifested by expressing OCT4/SOX2/NANOG associated programs) with their relaxed cell cycle checkpoints [91]. Such a state is often shared by aggressive primary cancers [92–95] and displayed by polyploidised cells of primary and established tumour cell lines resisting genotoxic stress [96–98]. The previously postulated cancer embryonic stem cell-like attractors [99–101] match between that of the two-cell embryo and that of onset of first lineage commitment [102]. As we have revealed here, physiological polyploidy in heart, liver, and placenta does not go that far in embryonalisation and genome destabilisation and therefore is separated from cancer.

## MATERIALS AND METHODS

### Data sources and pairwise cross-species comparative approach description

To reveal the evolutionary conserved and thus functionally important effects of polyploidy on c-MYC regulated features, we investigated the activity of the c-MYC interacting genes in homologous tissues of mammalian species differing by ploidy and in polyploid vs diploid cells of the same tissue. The comparative cross-species and intra-species approach is instructive because evolutionary distance enhances the signals by helping to distinguish them from noise emanating from species- and tissue-specific effects [103]. The multi-level signal-to-noise filtration is particularly precautious for investigation of polyploidy because polyploidy may exert only weak and idiosyncratic effects on gene expression because of preserving gene-dosage balance [51].

The approach of reciprocal cross-species comparison was developed and applied previously [34, 35, 104]. The data for the previous analysis were taken from Su et al. [105]. Since that time, the amount of annotated genes increased by more than 40% [1] and new bioinformatic approaches were developed, including the analysis of protein interactions. Altogether, these novelties allow us to step away from the conservative arbitrary 2–fold threshold for the expression difference that is appropriate only for analysis of strong effects and to accept the threshold of 15% that can be applied for evaluation of small fluctuations of gene expression [106, 107]. Finally, given that transcriptional regulators exhibit small expression amplitude, 15% expression difference may provide important information about the influence of polyploidy on transcription factors and chromatin regulators [106–108]. We performed the cross-species pairwise reciprocal comparison using the transcriptomic data for polyploid vs diploid organs, specifically, for human heart (polyploid) vs mouse heart (diploid) and mouse liver (polyploid) vs human liver (diploid). The transcriptomic data were from the database obtained from next generation sequencing (RNA-seq) by Brawand and colleagues, 2011 [109]. To increase the reliability of cross-species approach, we also analysed the genes selected from the microarray database [41] using the same cross-species reciprocal comparison algorithm. RNA-seq is more sensitive than microarrays [110], therefore the RNA-seq database was treated as a primary database, whereas the microarray data were considered as a secondary database providing additional support.

To understand similarities in ploidy-associated gene regulation at the inter- and intra-species levels, we compared the results of the human and mouse heart and liver analysis with the results obtained for purified 4n and 2n cells of early mouse decidua taken from the microarrays database [111].

### Data normalization

The analysis of RNA-seq data by Brawand and colleagues [109] was performed with the genes whose expression differed in the same direction with regard to ploidy in heart and in liver. In both comparisons the genes should have higher (or lower) expression in a polyploid tissue compared with a diploid tissue. Since we compared two different tissues in opposite directions in different species (human vs. mouse in the case of heart, and mouse vs. human in the case of liver), the effects of tissue-specificity and species-specificity were presumably removed. The same approach was applied for the microarray data by Wu and colleagues [41].

The one-to-one human-mouse orthologous genes were obtained from the Homologene database [112]. The expression levels of orthologous genes were analyzed using the 'limma' package specially developed for revealing differentially expressed genes in whole-transcriptome analyses [113]. Comparison of different software packages showed that limma is the method of choice for goals similar to those pursued in our work [114]. It is especially valuable that limma allows analyzing in a similar way both RNA-seq and microarray data [113]. The data were normalized with quantile normalization implemented in limma and the differential gene expression (with its statistical significance) was determined on the ground of among-samples variation within each tissue within each species using the modified t-test implemented in limma. The intra-species analysis of 4n vs. 2n decidual mouse cell transcriptomes was performed similarly. These transcriptomes were from the work by of Ma and colleagues [111].

Then, we selected the genes, which exhibited differential expression between polyploid and diploid tissues (cells) above 15%. As a result, we obtained three gene lists containing the genes that are common for heart, liver and decidua cells (Supplementary Table S1), for human heart and mouse liver (Supplementary Table S2), and for 4n vs. 2n early mouse decidua cells (Table 3). Then, these three gene lists were subjected to gene module enrichment analysis (see below the method description and Supplementary Tables S4, S5, S6).

To identify genes with maximal ploidy association, we matched the genes that are common for heart-liver and decidua cells (Supplementary Table S1) (i.e. were obtained using the databases [109, 111] with the gene lists for human and mouse heart and liver obtained using the data bases [41]. The resulted gene list is presented in Table 1.

### Principal component analysis

To find out, whether the results of cross-species gene-by-gene comparison can be confirmed by other bioinformatic approaches, we applied principal component analysis (PCA) to the raw data matrix having samples as variables and genes as statistical units.

The idea is to confirm the gene-by-gene *a priori* approach with a data-driven strategy, letting a ‘ploidy’ specific principal component to emerge from the data. The principal components are orthogonal each other by construction, the data-driven emergence of a ‘pure ploidy component’ distinct from ‘tissue’ and ‘species’ components is equivalent to an unsupervised normalization for tissue and species effects. The genes endowed with extreme scores on such a ‘ploidy’ component are the ‘image in light’ of tissue and species independent ploidy effect on transcription pattern.

For this propose we used microarray data [41] for human and mouse heart and liver. This approach enabled us to evaluate the impact of shared variable and species-specific, tissue-specific and ploidy-specific variables separately [52]. As a result, we obtained two lists of genes demonstrating statistically significant plody-asociated variation (not less 2 standard deviations) for human and mouse heart and liver (Supplementary Table S7) and for 4n/2n decidua cells (Supplementary Table S8). Then these gene lists were subject to gene module enrichment analysis and the modules that are regulated by ploidy in similar ways for heart-liver and placenta were identified (Supplementary Tables S9 and S10).

### Analysis of biological modules

To find out which biological modules were over-represented among the ploidy-associated genes, we applied a double control. We tested the genes from all three data sets (Supplementary Tables S4–S6) with higher and lower expression in polyploid vs diploid tissues and cells, respectively, for enrichment of Gene Ontology (GO) categories and molecular pathways with regard to all human-mouse orthologous genes (13965 genes) and simultaneously with regard to all known orthologous c-MYC – interacting genes (3734 genes). The enriched GO categories and molecular pathways were found using the hypergeometric distribution of probability (implemented in R package) as in the previous work [115, 116]. GO categories were taken from GO database [117]. For each GO category, all its subcategories were collected using Gene Ontology acyclic directed graphs, and a gene was regarded as belonging to a given category if it was mapped to any of its subcategories. As a source of molecular pathways, the NCBI BioSystems was used, which is a most complete compendium of molecular pathways from different databases [112]. The redundancy was removed by uniting entries with identical gene sets. The adjustment for multiple comparisons was done according to method by Storey and Tibshirani [118]. This procedure gives q-value, which can be considered as p-value corrected for multiple tests.

Significance levels were set at p<0.01 and q<0.15. We choosed these thresholds on the ground of recommentations of GSEA group and other authors [119, 120]. Protein-protein interactions were taken from the STRING database [1].

### Immunofluorescent and RT-PCR study

Immunofluorescent and RT-PCR study of polyploid versus diploid hepatocytes in adult IRC mice and Wistar rats were performed in three independent experiments. For details of the method, see Supplementary 1.

## SUPPLEMENTARY FIGURES AND TABLES






















