# Supplementary material for: Somatic polyploidy is associated with the upregulation of c-MYC interacting genes and EMT-like signature
Source: Oncotarget. 2016 Sep 19;7(46):75235–60. doi: 10.18632/oncotarget.12118 (PMC5342737; doi:10.18632/oncotarget.12118)
Supplement: Supplementary file 1 [file oncotarget-07-75235-s001.pdf]

Somatic polyploidy is associated with the upregulation of c-MYC interacting genes and EMT-like signature

SUPPLEMENTARY DATA

Materials and methods for Immunofluorescence and RT-PCR study of rodent liver

Experimental animals

Pathogen-free, female adult AKR mice between 6-8 weeks of age and Wistar rats (120-140g) were obtained from the Latvian Experimental Animal Laboratory of the Riga Stradins University and maintained under pathogen-free conditions in accordance with the principles and guidelines of the Latvian and European Community Laws. The experimental protocol was approved by the local Animal Protection Ethical Committee of the Latvian Food and Veterinary Service (permission no. 55/15.03.2013). Immunofluorescent and RT-PCR study of polyploid versus diploid hepatocytes in AKR mice was performed in three independent experiments; Wistar rats were examined by immunofluorescence in two experiments.

Immunofluorescence

Mouse or rat liver tissue imprints on polylysine-coated microscopic slides were fixed in methanol for 7 min at -20°C and dipped 10 times in ice cold acetone. Slides were then washed thrice in TBS 0.01% Tween 20 (TBST) for 5 min. Slides were subsequently blocked for 15 min in TBS, 0.05% Tween 20%, 1% BSA at room temperature. Samples were covered with TBS, 0.025% Tween 20%, 1% BSA containing primary antibody - c-Myc (Santa Cruz, sc-40) and GNL3 (Abcam, ab70346) and incubated overnight at 4°C in a humidified chamber. Samples were then washed thrice in TBST and covered with TBST containing the appropriate secondary antibodies (Goat anti-mouse IgG Alexa Fluor 488 (A31619, Invitrogen0) and Goat anti-rabbit- IgG Alexa Fluor 594 (A31631, Invitrogen)) and incubated for 40 min at room temperature in the dark. Slides were washed thrice for 5 min with TBST and once for 2 min in PBS. Samples were then counterstained with 0.25 µg/ml DAPI for 2 min, and finally embedded in Prolong Gold (Invitrogen).

Microscopy and immunofluorescence image cytometry

Slides were evaluated using a Leitz Ergolux L03-10 microscope equipped with Sony DXC 390P colour video camera, for microscopic observations; in addition to separate optical filters, a three-band BRG (blue, red, green) optical filter (Leica) was used. Image cytometry was carried out by semi-automatic measuring fluorescence values for each cell nuclei in all three channels and analysed using Image-Pro Plus 4.1 software (Media Cybernetics).

RT-PCR

Total RNA was extracted from mouse liver by using TRIZOL (Invitrogen). cDNA was synthesized using First Strand cDNA Synthesis Kit (Fermentas MBI) according to the protocols of the manufacturer. The absence of contamination with chromosomal DNA was verified by PCR using actin primers.

Amplification was carried out in a total volume of 50 µl with 1 µl of cDNA and followed primers: beta-actin F/R; mGNL3 F1/R1; mGNL3 F2/R2; m-myc F1/R1; m-myc F2/R2 under standard conditions using 1.0 unit of Taq polymerase (Thermo Scientific). Amplified PCR products were analyzed by 1% agarose gel.

Sequences of primers, length of fragments, and annealing temperatures used for RT-PCR

Gene/ Primer	Forward primer sequence 5'→3'	Reverse primer sequence 5'→3'	Length of fragment, bp	Anneal. temp., °C
β-actin	GGACTTCGAGCAAGAGATGG	AGCACTGTGTTGGCGTACAG	234/329	56
mGNL3 (1)	CATTGCCAGGAACTTAAAAAG	TTTGCTTTGGAATGGAACAAC	300	54
mGNL3 (2)	GCTCAAAGAAGAGGTCTGCAC	GTTAAATGAGGGCCCTTGAG	242	56
m-myc (1)	TCCTGTACCTCGTCCGATTC	GCACCTCTTGAGGACCAGTG	279	58
m-myc (2)	CTCAGTGGTCTTTCCCTAC	CTCGGGATGGAGATGAGC	283	54

SUPPLEMENTARY TABLES

Supplementary Table S1: MYC interacting genes that are induced or repressed in polyploid vs diploid human and mouse heart and liver and in 4n/2n decidua cells obtained by gene by gene transcriptome comparison

See Supplementary File 1

Supplementary Table S2: MYC interacting genes that are significantly induced or repressed in polyploid vs diploid human and mouse heart and liver obtained by cross species gene by gene comparison

See Supplementary File 2

Supplementary Table S3: Ploidy associated genes in c-Myc interactome of decidua 4n/2n cells obtained by gene by gene transcriptome comparison

See Supplementary File 3

Supplementary Table S4: Gene modules enriching for MYC interacting genes that are significantly induced or repressed in polyploid vs diploid human and mouse and mouse heart and liver and in 4n/2n decidua cells

See Supplementary File 4

Supplementary Table S5: Gene modules enriching for Myc interacting genes that that are induced in polyploid vs diploid human and mouse heart and liver

See Supplementary File 5

Supplementary Table S6: Gene modules enriching for Myc interacting genes that are significantly induced or repressed in 4n vs 2n decidua cells

See Supplementary File 6

Supplementary Table S7: MYC interacting genes that are significantly induced or repressed in polyploid vs diploid human and mouse heart and liver

See Supplementary File 7

Supplementary Table S8: Gene modules enriching for principal component analysis revealed genes that are induced or repressed in polyploid vs diploid heart and liver

See Supplementary File 8

Supplementary Table S9: MYC interacting genes that are significantly induced or repressed in 4n vs 2n decidua mouse cells

See Supplementary File 9

Supplementary Table S10: Gene modules enriching for PCA revealed genes that are induced in 4b/2n mouse decidua cells

See Supplementary File 10
